# A Different View on the Checkerboard? Alterations in Early and Late Visually Evoked EEG Potentials in Asperger Observers

**DOI:** 10.1371/journal.pone.0090993

**Published:** 2014-03-14

**Authors:** Juergen Kornmeier, Rike Wörner, Andreas Riedel, Michael Bach, Ludger Tebartz van Elst

**Affiliations:** 1 Institute for Frontier Areas of Psychology and Mental Health, Freiburg, Germany; 2 Eye Center, Albert-Ludwigs-University of Freiburg, Freiburg, Germany; 3 PPD Germany GmbH & Co Kg, Karlsruhe, Germany; 4 Section for Experimental Neuropsychiatry, Clinic for Psychiatry & Psychotherapy, Albert-Ludwigs-University of Freiburg, Freiburg, Germany; University College London, United Kingdom

## Abstract

**Background:**

Asperger Autism is a lifelong psychiatric condition with highly circumscribed interests and routines, problems in social cognition, verbal and nonverbal communication, and also perceptual abnormalities with sensory hypersensitivity. To objectify both lower-level visual and cognitive alterations we looked for differences in visual event-related potentials (EEG) between Asperger observers and matched controls while they observed simple checkerboard stimuli.

**Methods:**

In a balanced oddball paradigm checkerboards of two checksizes (0.6° and 1.2°) were presented with different frequencies. Participants counted the occurrence times of the rare fine or rare coarse checkerboards in different experimental conditions. We focused on early visual ERP differences as a function of checkerboard size and the classical P3b ERP component as an indicator of cognitive processing.

**Results:**

We found an early (100–200 ms after stimulus onset) occipital ERP effect of checkerboard size (dominant spatial frequency). This effect was weaker in the Asperger than in the control observers. Further a typical parietal/central oddball-P3b occurred at 500 ms with the rare checkerboards. The P3b showed a right-hemispheric lateralization, which was more prominent in Asperger than in control observers.

**Discussion:**

The difference in the early occipital ERP effect between the two groups may be a physiological marker of differences in the processing of small visual details in Asperger observers compared to normal controls. The stronger lateralization of the P3b in Asperger observers may indicate a stronger involvement of the right-hemispheric network of bottom-up attention. The lateralization of the P3b signal might be a compensatory consequence of the compromised early checksize effect. Higher-level analytical information processing units may need to compensate for difficulties in low-level signal analysis.

## Introduction

Patients with Autism Spectrum Disorder (ASD) are characterized by lifelong routines, circumscribed interests and deficits in social cognition and communication, e.g. [1]. The prevalence in the general population is estimated to be above 1% [2]. ASD results in significant socioeconomic consequences with up to 50,000 € annual costs per patient in particular due to secondary psychiatric problems and early retirement [3]. This illustrates the need for further etiological and therapeutic research.

### High Functioning Autism as a Possibly More Homogenous Autistic Subcategory

Traditionally, autism has been conceptualized as a severe form of neurodevelopmental disorder, which is associated with mental retardation, and severe deficits of intelligence and language in the majority of cases [4]. However, recent research has indicated that there is a broad variety of different severities and phenotypes of ASD including those with normal or even above average intelligence [5]. Secondary and syndromal forms of ASD which often go along with subnormal IQ and learning disabilities are increasingly distinguished from primary familial but probably not mono- or oligogenetic forms [4], [5], [6]. Theoretical considerations as well as clinical observations support the assumption that the subgroup of “primary” autism might more often be associated with normal or even above average intelligence scores [5], [7]. We thus concentrated on patients with Asperger syndrome (“AS”) with normal or above average IQ in order to get a more homogenous sample and thus to minimize the number of confounding factors [8].

So far clinical diagnostics have been mainly based on behavioral variables. Physiological markers are rare and related findings inconsistent, e.g. [9], [10], [11]. Further, the definitions of AS and ASD in general are primarily determined by cognitive, especially social symptoms whereas specificities in lower level sensory processing have only recently been integrated in the diagnostic criteria of DSM-V (www.dsm5.org). Still, it has long been recognized that such lower level perceptual and in particular visual abnormalities in autism might well be linked to the core pathophysiology of autism. Related reports range from abnormalities in the contribution of magnocellular pathways to face perception, e.g. [12], to alterations in processing of motion, e.g. [13], contrast, e.g. [14], or spatial frequency, e.g. [15]. Simmons et al. provided a comprehensive review about psychophysical and physiological indicators of altered visual processing in autistic observers [10].

The focus in the present EEG study was thus on the question of whether this higher visual sensitivity of Asperger observers for small object details may be visible in early visual stimulus-dependent EEG signatures and whether potential findings from lower-level processing correlate with EEG signatures related to higher-level/cognitive processing.

Spatially periodic stimuli like checkerboards are well-established visual stimuli in clinical electrodiagnostics (EEG), their reversal evoking a reliable modulation of early visual event related potential (ERP) amplitudes as a function of spatial frequency, e.g. [16]. In the present study we analyzed checkerboard onset ERPs and focused on the amplitude difference between the negative N2 component and the positive P2 component, e.g. [16]. The amplitude difference between N2 and P2 (sometimes also labeled as C2 and C3) is known to vary as a function of the stimulus’ size (or “dominant spatial frequency” in technical terms), with maximal values at intermediate spatial frequencies, e.g. [17], [18]. In the following sections we will call this amplitude modulation as a function of checksize the “*ERP checksize effect*”. In our experiment we presented checkerboards with two different checksizes and looked for differences in the ERP checksize effect between AS and control observers.

The second focus of the current study was on the P3b ERP component, which is well-known as a cognitive component and has recently been discussed in the context of conscious versus unconscious perception, e.g. [19]. The P3b typically occurs between 250 ms and 600 ms after onset of an infrequent task-relevant target stimulus or infrequent omissions of a periodical stimulus (so called “oddball paradigm”). Its amplitude is negatively correlated with the target stimulus’ frequency and positively correlated with stimulus’ discriminability. P3b latency and reaction times are negatively correlated with stimulus discriminability (for recent reviews see [20], [21]). The P3b is labeled as “cognitive” because its amplitude is modulated by the frequency and task-relevance of a stimulus, but not by the modulation of visual features (given a certain level of visibility). This behavior is in contrast to early “visual” ERP signatures that show amplitude and latency modulation as a function of lower-level stimulus features like luminance or size as in the present study but typically not as a function of the task.

In a balanced oddball paradigm we presented fine and coarse checkerboard stimuli both as rare targets and frequent non-targets in separate experimental runs and compared P3b amplitudes and latencies between AS and control participants. We further asked whether a potential lower-level modulation of the ERP checksize effect in AS participants would correlate with a higher-level P3b amplitude and/or latency modulation.

## Methods

### Participants

21 Asperger (AS) participants and 17 healthy control participants were tested in this EEG-study. Control participants were selected to match the AS participants in age (±3 years) and gender. All participants had German school education comparable to junior high school or high school. Due to technical reasons only 19 AS participants (mean age = 41.3, SD = 10.7; 6 females) and 16 controls (mean age = 38.8, SD = 11.5, 6 females) entered the analysis. This resulted in 13 matched pairs (4 female) of AS observers (mean age: 39 years, SD = 10.6) and control observers (mean age 38.3, SD = 10.9).

All participants completed the autism-spectrum questionnaire “AQ” [22] and the empathy questionnaire “EQ” [23]. In the AQ, AS observers scored above 34 (Mean = 43.1; SD = 5) and the control observers scored below 28 (Mean = 15.1; SD = 5.5). The EQ scores showed the reverse picture – high scores in the control group (Mean = 43.3; SD = 8.2; Min = 29) and low scores in the AS group (Mean = 14.2; SD = 6.3; Max = 28).

All participants had a normal visual acuity. All participants gave their informed written consent. The study was performed in accordance with the ethical standards laid down in the Declaration of Helsinki [24] and was approved by the ethics board of the Albert-Ludwigs-Universität Freiburg, Germany.

### Clinical Diagnostics

At the Division of Psychiatry and Psychotherapy, University Medical Center Freiburg, the clinical diagnosis of autism spectrum disorders and AS is established as a consensus diagnosis of a multiprofessional team following the recommendations of the draft version of the NICE guidelines (National Institute for Health and Clinical Excellence: Autism in Adults: full guideline DRAFT (December 2011; http://www.nice.org.uk/nicemedia/live/12339/57402/57402-.pdf)). According to these guidelines “a number of key components […] should form the basis of any comprehensive assessment of an adult with possible autism, as follows: the core symptoms of autism include social interaction, communication and stereotypical behavior; a developmental history spanning childhood, adolescence and adult life; the impact on current functioning including personal and social functioning, educational attainment and employment” (NICE 2012 page 134/135). At the center named above, the diagnostic principles are realized in a structured way. The clinical diagnosis includes a thorough history of the patient following the above principles, a history of carriers (parents, partners, siblings etc.) and behavioral observations in a diagnostic process that usually takes several sessions. Psychometric tools like AQ [19], EQ [20], Australian Scale for Asperger’s Syndrome (ASAS) [32], SRS [22], BVAQ [33], AAA [34]), and BDI [35] are obtained in a routine procedure prior to clinical assessment and are used also for differential diagnostics. Additionally, instruments like ADI-R [36] and ADOS [37] are applied in selected and unclear cases. The same is true for additional neuropsychological tests assessing executive and theory-of-mind capacities. The multiprofessional diagnostic team consists of three experienced senior consultant psychiatrists and two fully qualified senior psychologists. The final consensus diagnosis is made by all persons involved in the diagnostic process, which will invariably include at least two experienced consultant psychiatrists or psychologists.

### Stimuli

The stimuli consisted of fine and coarse checkerboards with checksizes of 0.6° and 1.2° visual angle and a grey screen following each checkerboard presentation. Checkerboards and grey screen subtended a field of 13.25° (width)×14.25° (height) visual angle. Luminance of the white and black checks was 220 cd/m^3^ and 1.55 cd/m^3^. Luminance of the grey screen was 110 cd/m^3^.

### Experimental Paradigm

**Figure 1:**
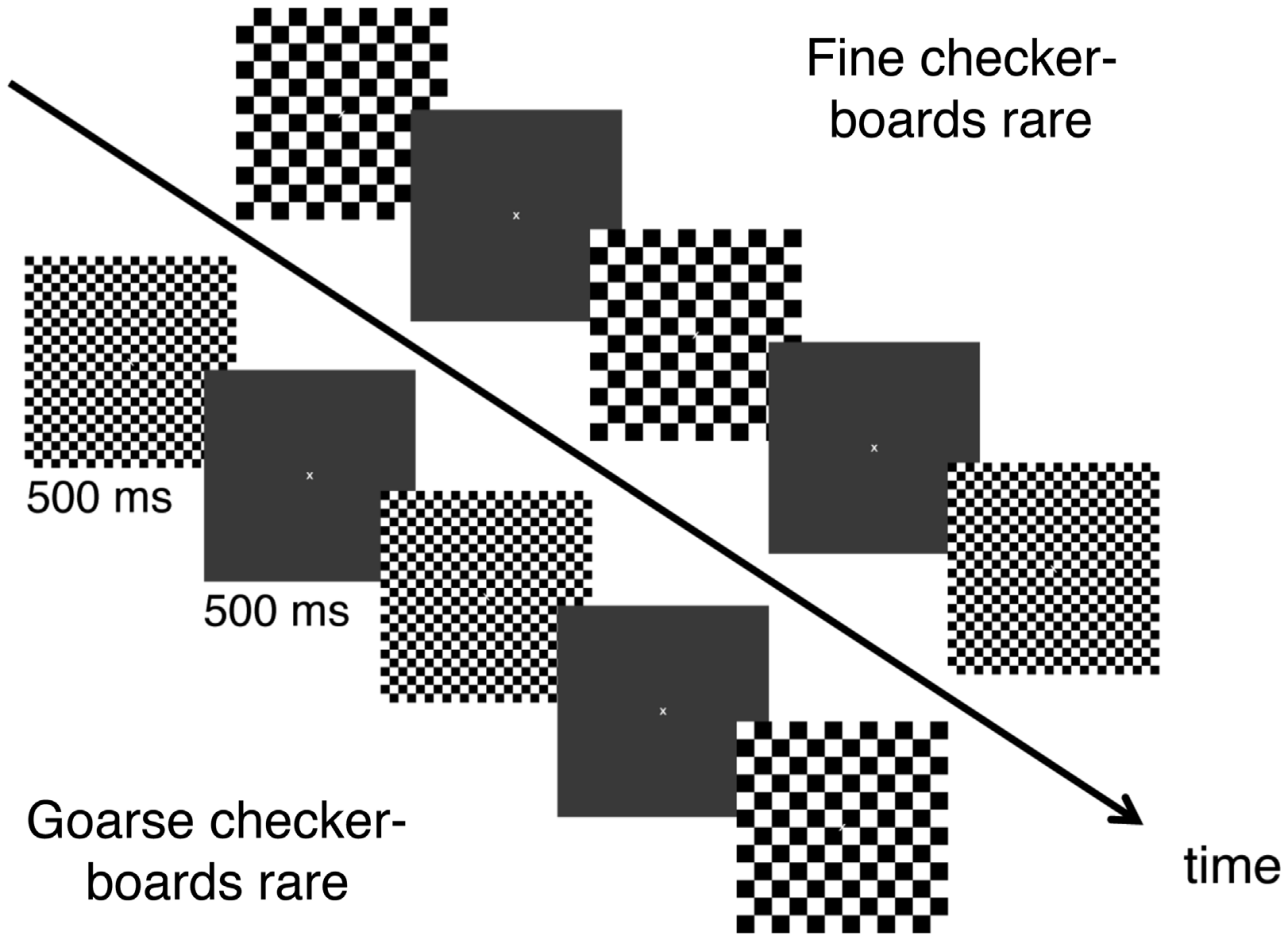
Experimental Paradigm. During one experimental block, checkerboards with two different checksizes were presented in random order with different frequencies (20% and 80%). Each checkerboard was presented for 500 ms and was followed by a grey screen for 500 ms. Participants had to count the occurrences of the rare checkerboards and to report the final number at the end of each experimental block.

The checkerboards were presented for 500 ms in an oddball paradigm, where rare stimuli occurred pseudo-randomly with a probability of p = 0.2. Each checkerboard was followed by a grey screen for 500 ms. From one checkerboard presentation to the next dark and white checks were exchanged (“reversal with interleaved grey”, Fig. 1). Participants were instructed to count occurrences of the rare target checkerboards, ignore the frequent non-target checkerboards as well as the grey screens and to fixate a central fixation cross which was continuously present. The experiment consisted of two conditions with either the fine checkerboards or the coarse checkerboards as rare target stimuli. Each of the two conditions (fine checkerboard rare and coarse checkerboard rare) consisted of 240 checkerboard presentations over four minutes with 80% (192) frequent and 20% (48) rare checkerboard sizes in a pseudo-randomized order.

The stimuli were generated by a Mac mini (1.5 GHz Power PC G4) and presented on a Philips Monitor GD 402 monochrome CRT screen with a refresh rate of 85 Hz. A control screen for the investigator was placed outside the experimental room.

### EEG Measurement and Processing

EEG signals were measured with the “Brain Vision” EEG system and referenced to a central midline electrode. Electrode locations were based on the extended 10–20 system [25]. Impedances were below 10 kΩ. The EEG signals were amplified with a factor of 1000, digitalized with a sampling rate of 500 Hz and streamed to disc.

Offline pre-processing of the EEG data included re-referencing to averaged mastoid electrodes and removal of single EEG trials containing artificial amplitude excursions above ±150 µV.

Currently we have no established repository for data related to the clinical electrophysiology. We will therefore archive the data locally and make them available to other researchers upon request together with information about the file format. This will be facilitated by keeping the EEG data and associated metadata strictly separate from any personal data that would make identification of the participants possible.

### Data Analysis

For each participant the checkerboard EEG trials were sorted with respect to stimulus size (spatial frequency) and stimulus frequency and selectively averaged to ERPs. The ERPs were digitally filtered with a latency-neutral low-pass filter with a cut-off at 25 Hz. Table 1 lists the minimal, maximal and mean number of trials per condition entering the ERP calculation.

**Table 1:** Number of averaged EEG trials.

	Asperger Observers				Controls			
	Fine Cb		Coarse Cb		Fine Cb		Coarse Cb	
	Freq	Rare	Freq	Rare	Freq	Rare	Freq	Rare
**Mean**	175.7	33.6	167.1	39.6	168.4	35.8	159.9	38.8
**SD**	26	1.1	46.8	7.2	30.4	12.2	42.5	8.3
**Max**	211	49	205	49	194	50	203	46
**Min**	92	10	53	17	102	6	53	38.8

Number of EEG trials per condition and observer group (SD = standard deviation) that entered ERP averaging. Cb: checkerboard; Freq: frequent Cbs.

#### Analysis of the ERP checksize effect

For each participant and channel we calculated the difference ERP traces (dERPs) between the *fine and coarse checkerboard ERPs* separately for the rare target stimuli and the frequent non-target stimuli, resulting in rare and frequent dERPs. This calculation isolated the *ERP checksize effect*, i.e. the ERP difference between fine and coarse checkerboards and reduced the inter-individual variance. We determined the amplitude of the ERP checksize effect for each participant as the amplitude difference between the maximal negative excursion in a predefined temporal region of interest (ROI) between 60 ms and 250 ms after checkerboard onset and the subsequent positive excursion for each electrode of a predefined spatial ROI involving the five occipital (PO9, O1, Oz, O2, PO10) and the five parietal (P7, P3, Pz, P4, P8) electrodes. Amplitude and latency of the ERP checksize effect were then analyzed with a mixed model ANOVA with the within factors TASK (2 steps: rare targets vs. frequent non-targets), CHANNEL (10) and HEMISPHERE (2 steps: left- vs. right-hemispheric electrodes) and the between factor GROUP (2 steps: AS vs. control observers) where all 35 participants entered. The factor SIZE, reflecting the different checksizes, disappeared by calculating the ERP difference traces as described above.

Since our results indicated also differences in amplitude variance between Asperger and control observers we also calculated post-hoc Barlett-Tests for homogeneity of variance.

### Analysis of the ERP P3b Effect

For each participant and channel we calculated the difference ERP traces between the rare and frequent checkerboard ERPs, separately for the fine and coarse checkerboards, in order to isolate the ERP oddball P3b and reduce the inter-individual variance. We determined the P3b amplitudes and latencies for each individual participant from the peaks in a temporal ROI between 250 ms and 600 ms after checkerboard onset for each electrode of our predefined spatial ROI involving three parietal (P3, Pz, P4), three central (C3, Cz, C4) and three frontal (F3, Fz, F4) electrodes.

Amplitude and latency of the P3b effect were then analyzed in a mixed model ANOVA with the within factors SIZE (2, fine vs. coarse checkerboards), CHANNEL (9) and HEMISPHERE (2, left- vs. right-hemispheric electrodes) and the between factor GROUP (2, AS vs. control observers).

Notice that the factor TASK, reflecting the frequent non-target stimuli and rare target-stimuli disappeared by calculating the ERP difference traces between frequent and rare stimuli, as described above. Where necessary, we conducted post-hoc randomization tests [26].

Finally we calculated Receiver Operating Characteristics (ROC) separately for the two effects and for their linear combination. A ROC curve displays the performance of a binary classifier. It depicts the classifiers’ sensitivity (proportion of correctly classified positives) and specificity (proportion of correctly classified negatives) as a function of changing output threshold [27]. We calculated the area under the ROC curve (“AUC”, in % of the maximal possible area) as a measure for the predictive power of the respective effect. An AUC of 50% indicates no discriminatory power, whereas 100% indicates optimal discriminatory power. In this case, the classifier would detect all true but no false positives.

In order to calculate the discriminatory power of the combination of the two effects we first calculated z-transformations for both data sets (the amplitude values of the checksize effect for the frequent checkerboards at electrode O1 and the P3b amplitude differences between electrode C4 and C3). We then added the P3b z-scores to the checksize effect z-scores and calculated a ROC analysis on these linear combination values. For a similar approach see [28].

## Results

### Psychophysical Results

The hit rate of the counting task was above 98% for both AS observers and normal controls without any group difference (p = 0.41 and p = 0.56 for fine and goarse checkerboards, based on permutation tests [26]).

### ERP Checksize Effect

**Figure 2:**
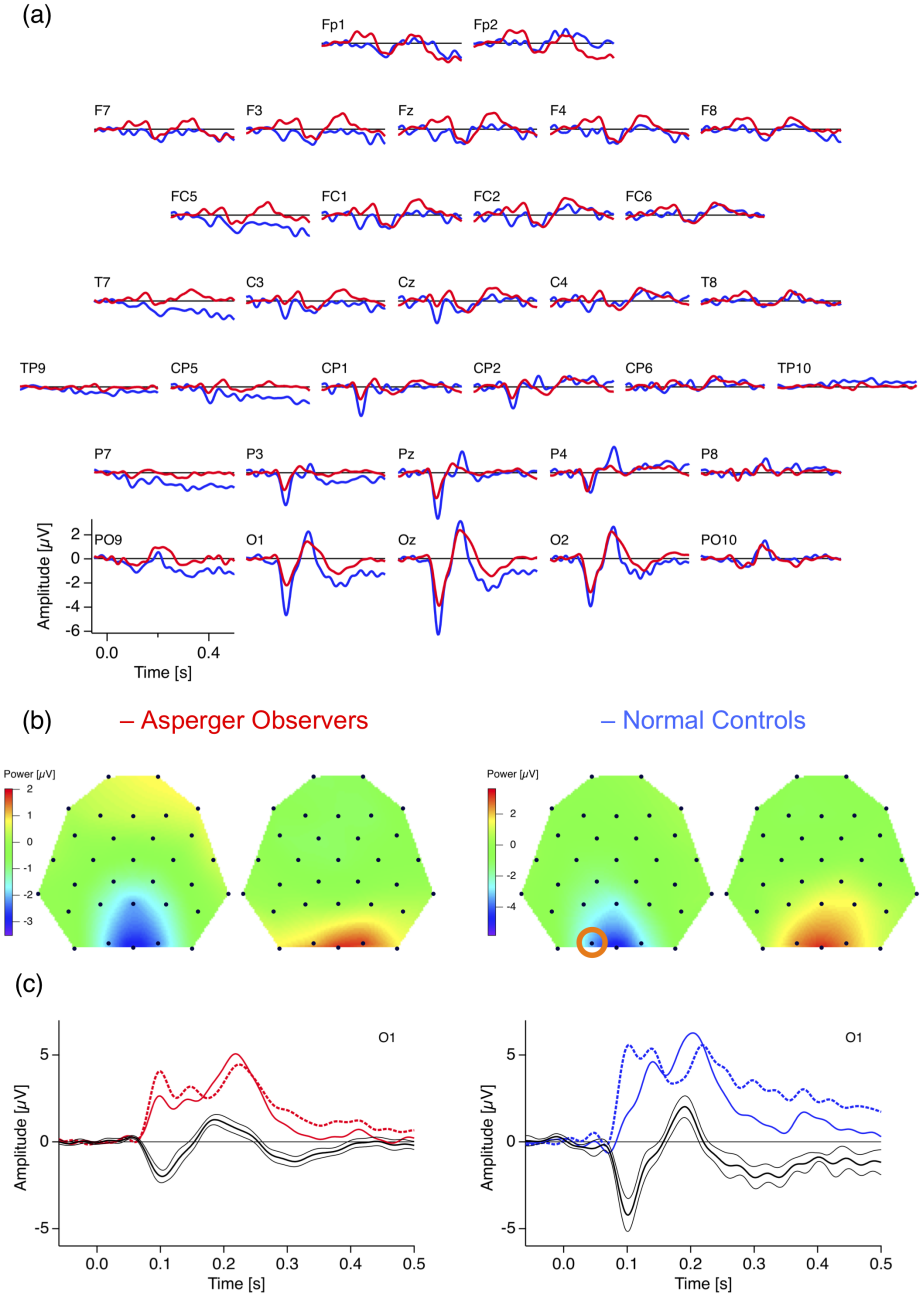
ERP checksize effect. (a) Grand mean ERP difference traces (fine minus coarse checkerboards) from AS observers (red) and controls (blue). Largest effects (negativity at 100 ms and positivity at 200 ms) occur at occipital electrodes. (b) Voltage maps with the spatial distribution of both the negativity (first and third voltage maps from left) and the positivity (second and fourth voltage map from left). Notice different scaling of the voltage maps between observer groups (c) Enlarged difference traces from the O1 electrode (indicated by the orange circle in (b)) ± SEM together with the underlying raw grand mean ERP traces (continuous lines for fine and dashed lines for coarse checkerboards).

**Figure 3:**
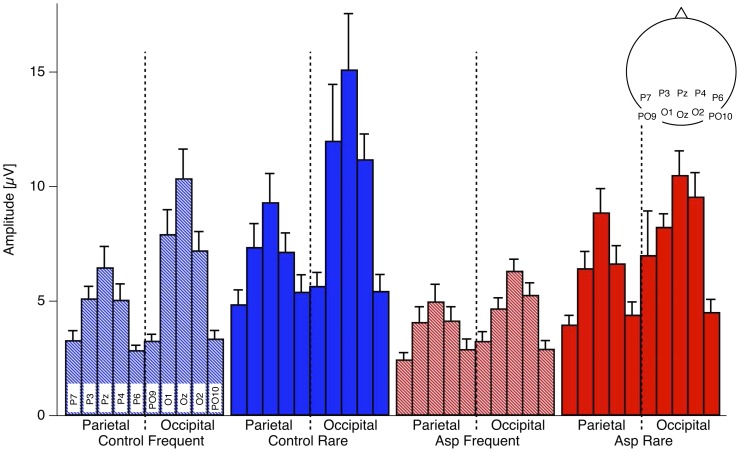
Grand Mean amplitudes (+SEM) of the individual checksize effect values at selected occipital and parietal electrodes (see scalp schema on the right top), separately for frequent non-target checker-boards (light colors) and rare target checkerboards (dark colors) and for AS (red) and control observers (blue). Dominance of midline occipital electrodes and smaller amplitudes for AS compared to Control observers can be observed.

Figure 2a shows the grand mean target- (light colors) and non-target- (dark colors) dERPs (fine minus coarse checkerboards) from both AS (red) and control observers (blue) for all 32 electrodes, arrayed according to their position on the scalp. A prominent ERP checksize effect can be observed at the occipital and parietal electrodes (see also the voltage maps in Fig. 2b). It consists of a sharp negative deflection at about 100 ms after stimulus onset and a subsequent sharp positive excursion roughly 100 ms later. Amplitudes of both components are larger for fine than for coarse checkerboards. Figure 2c shows the underlying raw ERP traces (continuous traces for fine and dashed traces for coarse checkerboards) together with the resulting differences (black traces, fine minus coarse checkerboards ± SEM) at the electrode O1 for AS (left, red) and control (right, blue) observers. Figure 3 shows the grand means of the individual amplitudes at parietal and central electrodes separately for AS and control observers and for frequent and rare checkerboards.

For the variable amplitude, the mixed-model ANOVA indicates (1) a strong effect of the factor GROUP (F(1,670) = 19.99, p = 9.2*10^−06^), reflecting the difference between Asperger and control observers, (2) a strong effect of the factor TASK (F(1,670) = 88.11, p = 2.2*10^−16^) reflecting larger amplitudes for the rare target checkerboards compared to frequent non-targets, and (3) a strong effect of the factor CHANNEL (F(9,670) = 34.36, p = 2.2*10^−16^) reflecting the dominance of the occipital midline electrodes, as can be seen in Fig. 3. The mixed-model ANOVA indicates further (4) an interaction between the factors GROUP and CHANNEL (F(9,670) = 1.95, p = 0.043) reflecting a larger difference of the ERP checksize effect between observer groups at central electrodes (Figs. 2 and 3). Uncorrected Post-hoc randomization tests [26] of differences between Asperger and control observers for frequent checkerboards are listed in Table 2. After Bonferroni-Holm correction for multiple testing [29] significance remained for the left occipital O1 electrode with p = 0.037.

**Table 2:** Checksize-effect: Post-hoc permutation tests.

Electrodes	p-values	Cohen’s D
P7	0.017	0.7
P3	0.11	0.43
Pz	0.11	0.43
P4	0.21	0.29
P8	0.35	0.2
PO9	0.17	0.32
**O1**	**0.0038** *	**1**
Oz	0.011	0.81
O2	0.037	0.63
PO10	0.2	0.32

Post-hoc permutation tests of diffe-rences in the checksize effect for the frequent checkerboards between Asperger and control observers.

*indicates re-maining significance after Bonferroni-Holm correction.

**Figure 4:**
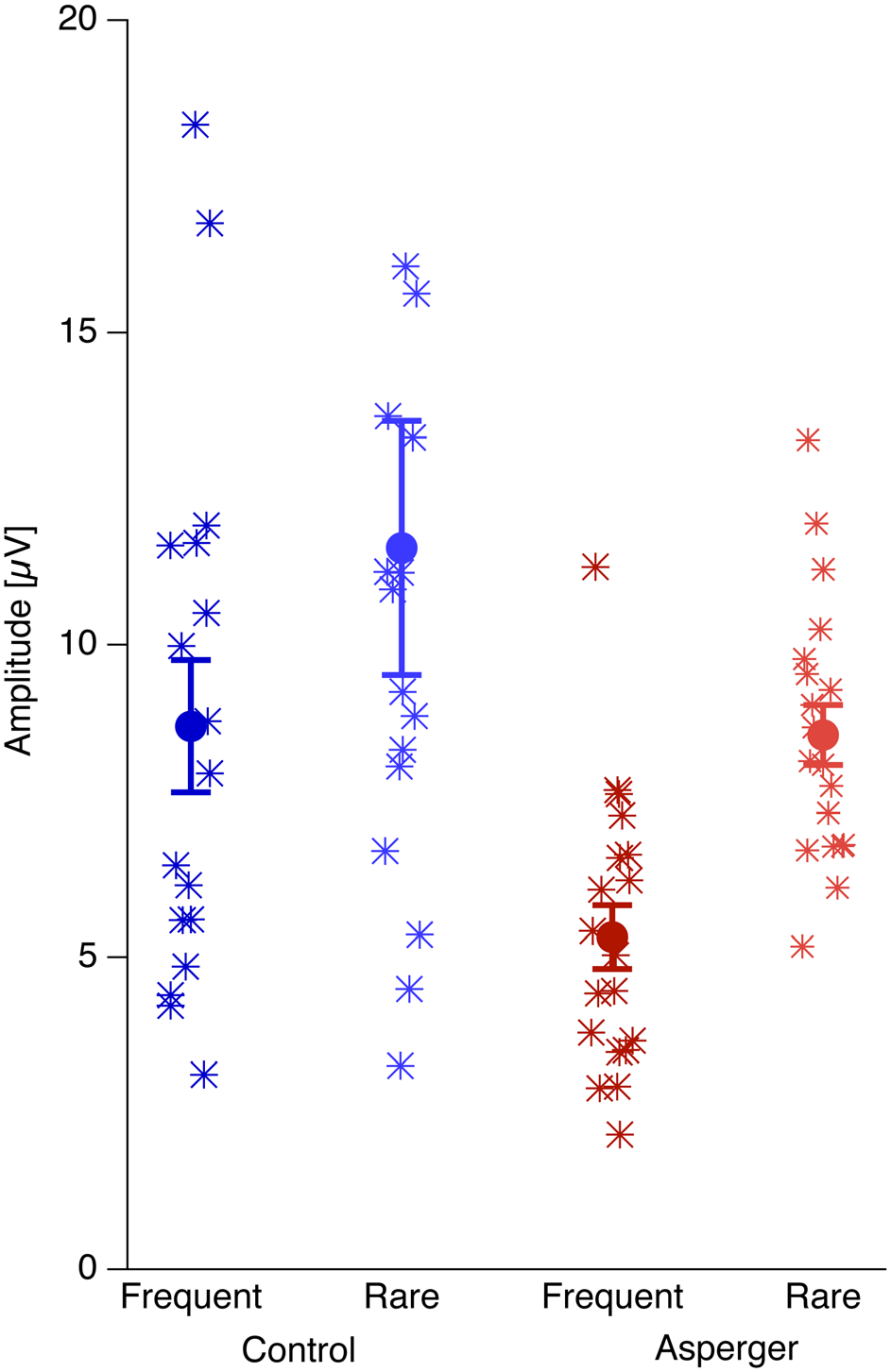
Individual (stars) and grand mean checksize effect data (circles ± SEM) at the right occipital O1 electrode. Here, the dark colors represent data from the frequent checkerboards and the light colors data from the rare checkerboards. Red colors represent data from the Asperger observers and blue colors represent data from the control observers. The data from the Asperger observers show both lower values and lower variance than those from the control observers.

Fig. 4 shows individual (stars) and grand mean data (circles ± SEM) at the right occipital O1 electrode. Here, the dark colors represent data from the frequent checkerboards and the light colors data from the rare checkerboards. Red colors represent data from the Asperger observers and blue colors represent data from the control observers. The data from the Asperger observers show both lower values and lower variance than those from the control observers (Barlett’s Test for equal variances: p<0.01 for the frequent checkerboards and p<0.05 for the rare checkerboards).

**Figure 5:**
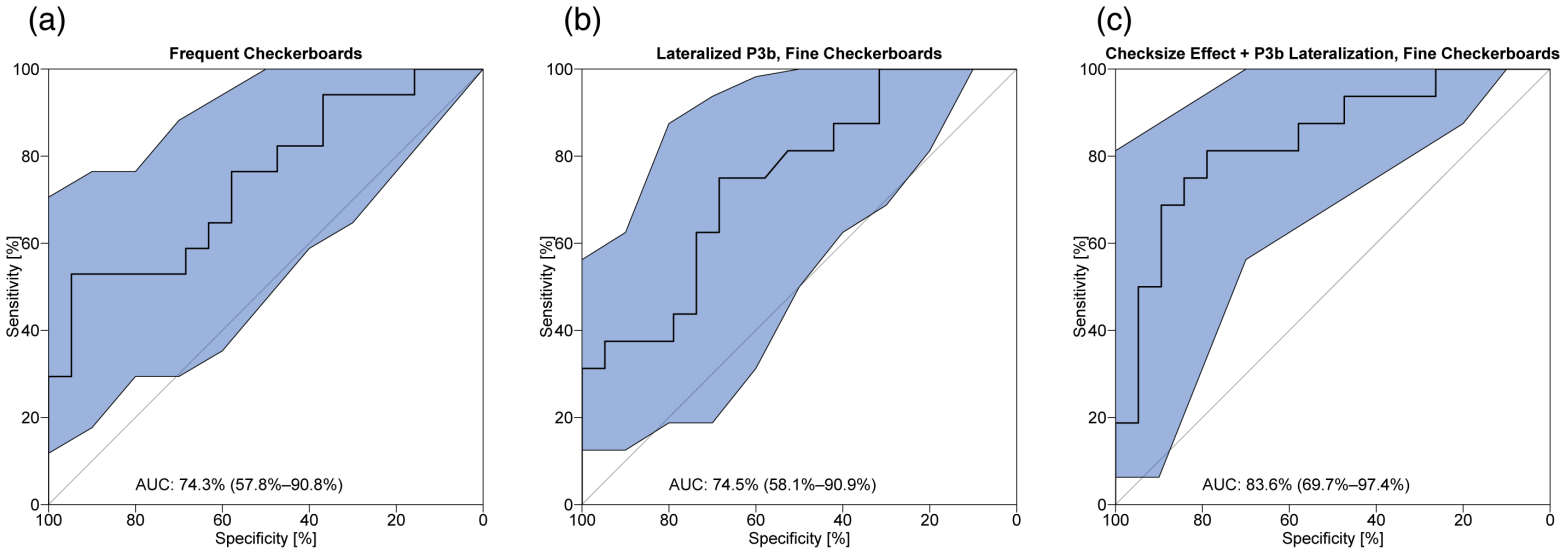
ROC curves of (a) the ERP checksize effect (b) the P3b lateralization effect and (c) a linear combination of both effects. The blue shaded areas indicate 90% confidence intervals based on bootstrap calculations (N = 10.000). AUC = area under curve (±90% confidence interval).

Figure 5a depicts the receiver operating characteristic (ROC) of the checksize effect for the frequent checkerboards. The area under the ROC curve can be regarded as the probability that the checksize effect identifies a randomly chosen participant correctly as either an Asperger or a Non-Asperger person and its value is 74.3% (57.8% –90.8% for a 95%-confidence interval).

### P3b Effect

**Figure 6:**
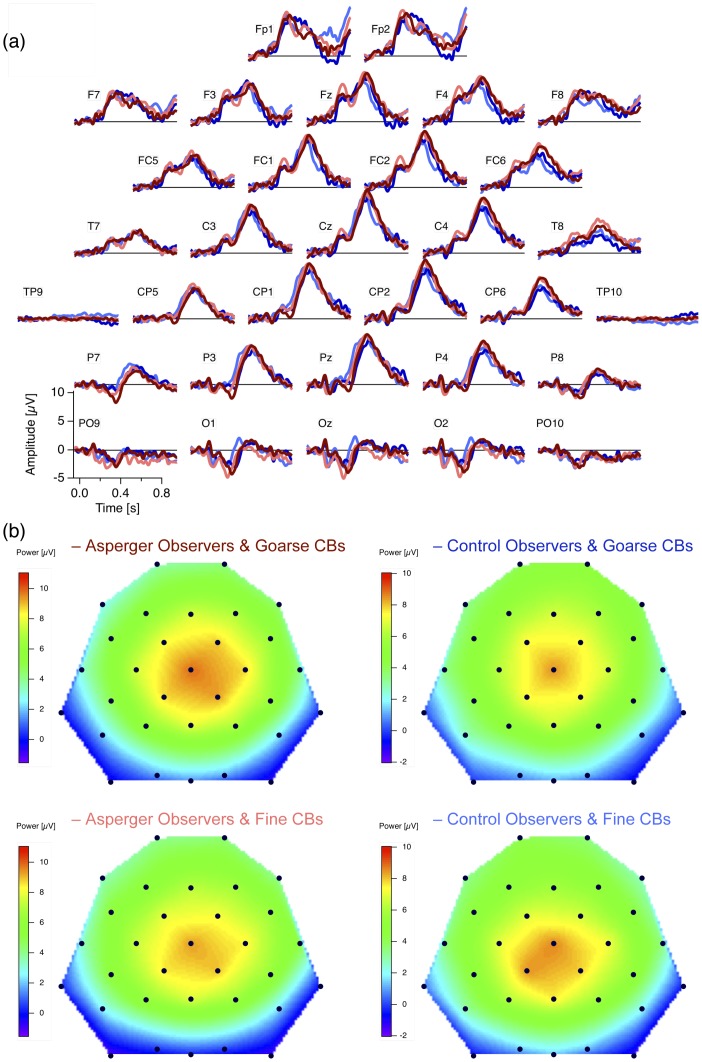
P3b lateralization effect. (a) Grand mean difference traces (dERPs, rare minus frequent checkerboards) separated for the AS (red) and control observers (blue) and for coarse (dark colors) and fine (light colors) checkerboards. (b) Voltage maps of the P3b 500 ms after stimulus onset. AS observers (left voltage maps) show similar amplitude and latency but a stronger right-hemispheric lateralization of the P3b at the central electrodes compared to control observers (right voltage maps).

Figure 5a shows the grand mean dERPs (rare minus frequent) for the fine (light colors) and coarse (dark colors) checkerboards from both AS (red) and control observers (blue) for all 32 electrodes, arrayed according to their position on the scalp. A huge oddball P3b can be observed roughly 500 ms after checkerboard onset with maximal amplitudes at parietal and central midline electrodes (see also the voltage maps in Fig. 6b). For the variable amplitude, the mixed model ANOVA indicates a significant effect for the factor CHANNEL (F(8,264) = 11.6, p = 4.2*10^−14^), reflecting the parietal and central midline dominance of the P3b effect as indicated by the voltage maps in Fig. 6b. The ANOVA further indicates an effect for the factor HEMISPHERE (F(1,33) = 9.7, p = 0.0038), with a slight right-hemispheric dominance of the P3b. This hemispheric asymmetry is more pronounced in AS observers than in controls, which is reflected in an interaction between GROUP and HEMISPHERE (F(1,33) = 8.3, p = 0.007) and which is also indicated in the voltage maps (Fig. 6b).

Table 3 provides the uncorrected results from post-hoc randomization tests. We collapsed the data from the two different checkerboard sizes and compared amplitudes from left-hemispheric and right-hemispheric electrodes at parietal, central and frontal sites. After Bonferroni-Holm correction for multiple testing [29] the central and frontal electrode pairs from the Asperger observers showed a significant right-hemispheric lateralization of the P3b (p = 0.005 and p = 0.006). No significant P3b-lateralization remained in the control observers.

**Table 3:** P3b post-hoc permutation tests.

	Asperger observers		control observers	
Electrodes	p-values	Cohen’s D	p-values	Cohen’s D
**P3 vs. P4**	0.006	0.25	0.16	0.13
**C3 vs. C4**	**0.001** *	0.46	0.39	0.031
**F3 vs. F4**	**0.001** *	0.35	0.27	0.076

p-value results from post-hoc permutation tests to compare the P3b amplitudes between hemispheres separately for Asperger and control observers.

*indicates remaining significance after Bonferroni-Holm correction.

**Figure 7:**
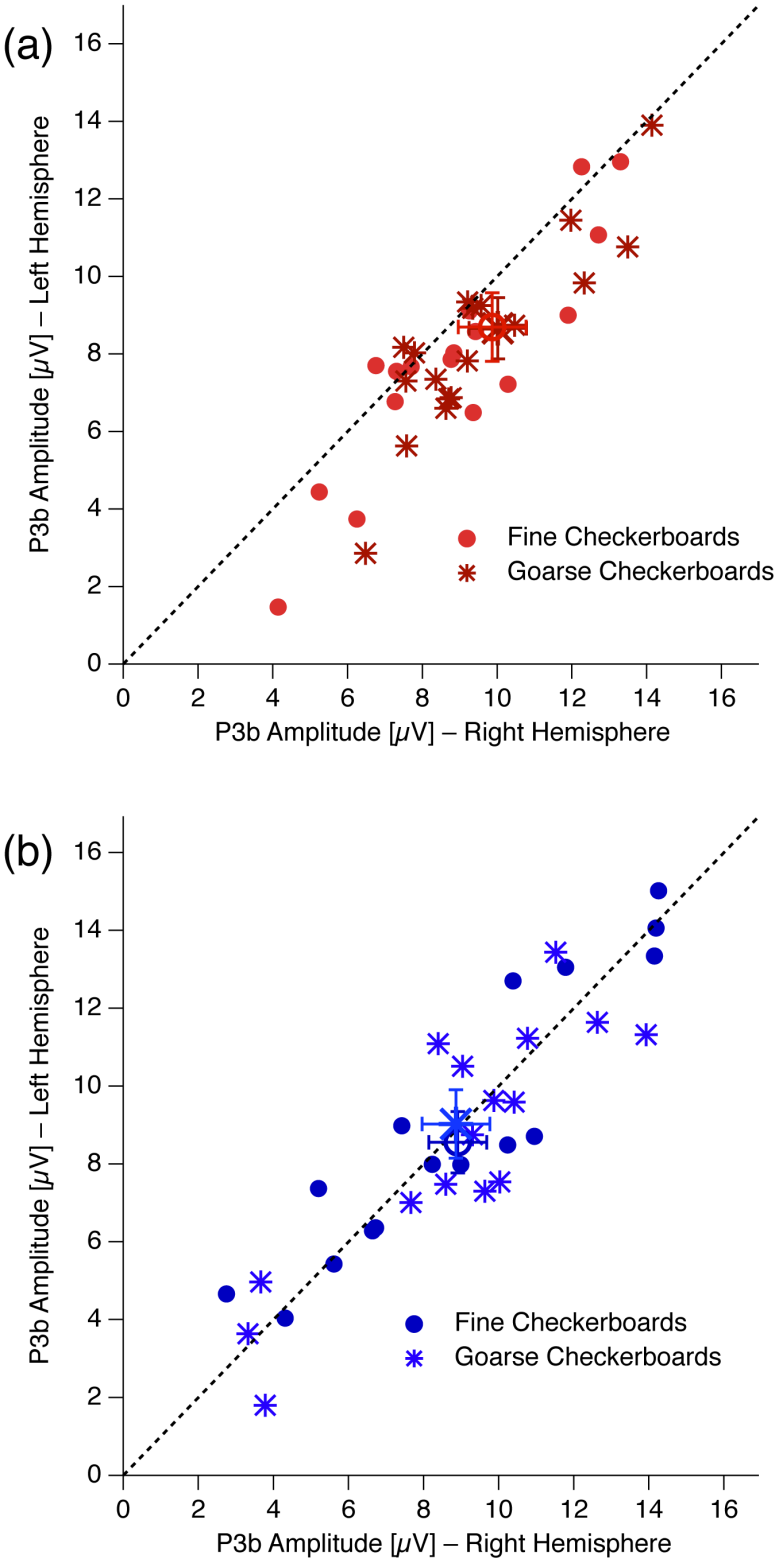
Individual P3b data to demonstrate the difference in hemispheric asymmetry of the P3b between Asperger (Fig. 7a) and control observers (Fig. 7b). Each individual icon represents amplitude values from the C3 electrode (left central hemisphere) on the ordinate and respective values from the C4 electrode (right central hemisphere) on the abscissa. Asymmetric distributions of icons, away from the bisection line indicate an asymmetric distribution of the P3b on the scalp. As can be easily seen, the data from the Asperger observers are much more lateralized to the right hemisphere (below the bisection line) than the control observer data. Stars (dark colors) and circles (light colors) represent data from the coarse and fine checkerboards respectively. Open circles and the larger stars represent the respective grand mean values (± SEM).

Fig. 7 shows individual P3b data to demonstrate the difference in hemispheric asymmetry of the P3b between Asperger (Fig. 7a) and control observers (Fig. 7b). Each individual icon represents amplitude values from the C3 electrode (left central hemisphere) on the ordinate and respective values from the C4 electrode (right central hemisphere) on the abscissa. Asymmetric distributions of icons, away from the bisection line indicate an asymmetric distribution of the P3b on the scalp. As can be seen easily, the data from the Asperger observers are much more lateralized to the right hemisphere than the control observer’s data. Circles (dark colors) and stars (light colors) represent data from the coarse and fine checkerboards respectively. Open circles and the larger stars represent the respective grand mean values (± SEM).

Figure 5b depicts the receiver operating characteristic (ROC) of the P3b effect for the frequent checkerboards. The area under the ROC curve can be regarded as the probability that the P3b effect identifies a randomly chosen participant correctly as either an Asperger or a Non-Asperger person and its value is 74.3% (57.8% –90.8% confidence interval).

Figure 5c depicts the ROC curve of the linear combination of both effects (Checksize effect+P3b effect). Discriminatory power increases to 83.6% (69.7% –97.4% for a 95%-confidence interval).

## Discussion

In the current EEG study we presented checkerboards with two different checksizes in a classical oddball paradigm and compared both the low-level visual ERP checksize effect and the higher-level oddball P3b between AS observers and matched controls. We found a smaller checksize ERP effect and smaller amplitude variance for AS than for control observers at occipital electrodes. This effect showed a discriminatory power of roughly 74%. We further found a right-hemispheric lateralization of the oddball P3b, which was more prominent in the AS than in the control observers. This P3b lateralization showed a discriminatory power of about 75%. A linear combination of both effects increased the discriminatory power to about 84%.

### The Early ERP Checksize Effect

In the last few years a number of studies focused on differences in EEG correlates of early visual processing between AS observers and controls. Milne et al. [15] presented sinusoidal gratings of different spatial frequencies (from 0.5 cpd [ = cycles per degree] to 8 cpd) in the pattern-onset mode [16] to autistic children and matched controls. They analyzed the EEG data with an independent component analysis and compared the identified components between autistic participants and matched controls. Some striate and extrastriate components around 100 ms after stimulus onset showed smaller power in autistic children than in controls. Boeschoten et al. [30] presented horizontal square-wave gratings and compared early visual ERP effects of varying spatial frequency (0.75 cpd –6 cpd) between autistic and control children. They found weaker effects on the N80 and P130 peaks in the autistic children compared to the matched controls. Jemel et al. [31] presented vertical sinusoidal gratings in a pattern-reversal mode with varying spatial frequencies and contrasts to adult ASD observers and matched controls. They found differences in the contrast tuning functions of the N80 and P100 for mid (2.8 cpd) and high (8 cpd) spatial frequencies. Most interestingly for the current study, their data indicate a smaller difference between their low and medium spatial frequency stimuli in ASD observers compared to controls (see their Figs. 2C and 3C).

The studies cited above differ in various parameters, like participants’ age, stimulus type, presentation mode, stimulus luminance, range of spatial frequencies, and the type of the focused EEG variables. Despite these differences all studies found weaker spatial frequency effects for ASD observers compared to control observers in early visual areas between 80 ms and 130 ms after stimulus onset.

EEG effects of spatial frequency from visual areas are well known in the literature and even belong to the standard diagnostic tools in clinical diagnostics, e.g. [16]. The timing of those EEG effects strongly depend on the range of spatial frequencies used. Early visual effects (N80 and P130) are known to be large with high spatial frequencies between 4 and 15 cpd, whereas strongest effects in the range of 200 ms after stimulus onset occur with intermediate values between 1 and 2 cpd (see for example Figure 5 in [18]). The studies cited above reported exclusively on early differences in spatial frequency EEG effects between AS and control observers. Our data show that similar differences are also present later, in the 200-ms-range.

Some authors tried to infer basic differences in early visual processing steps between observers with autism and controls from specific ERP components that are affected by the stimulus’ spatial frequency, i.e. whether the N80 or the P130 is more affected, e.g. [30], [31]. Such interpretations are fascinating; they stimulate more focused follow-up experiments and they may help replacing pure self-reported, phenomenological description of the perceptual differences between autistic and normal observers by objectively measurable signals and possibly pathophysiological description and understanding of this mental perceptual phenomenon.

**Figure 8:**
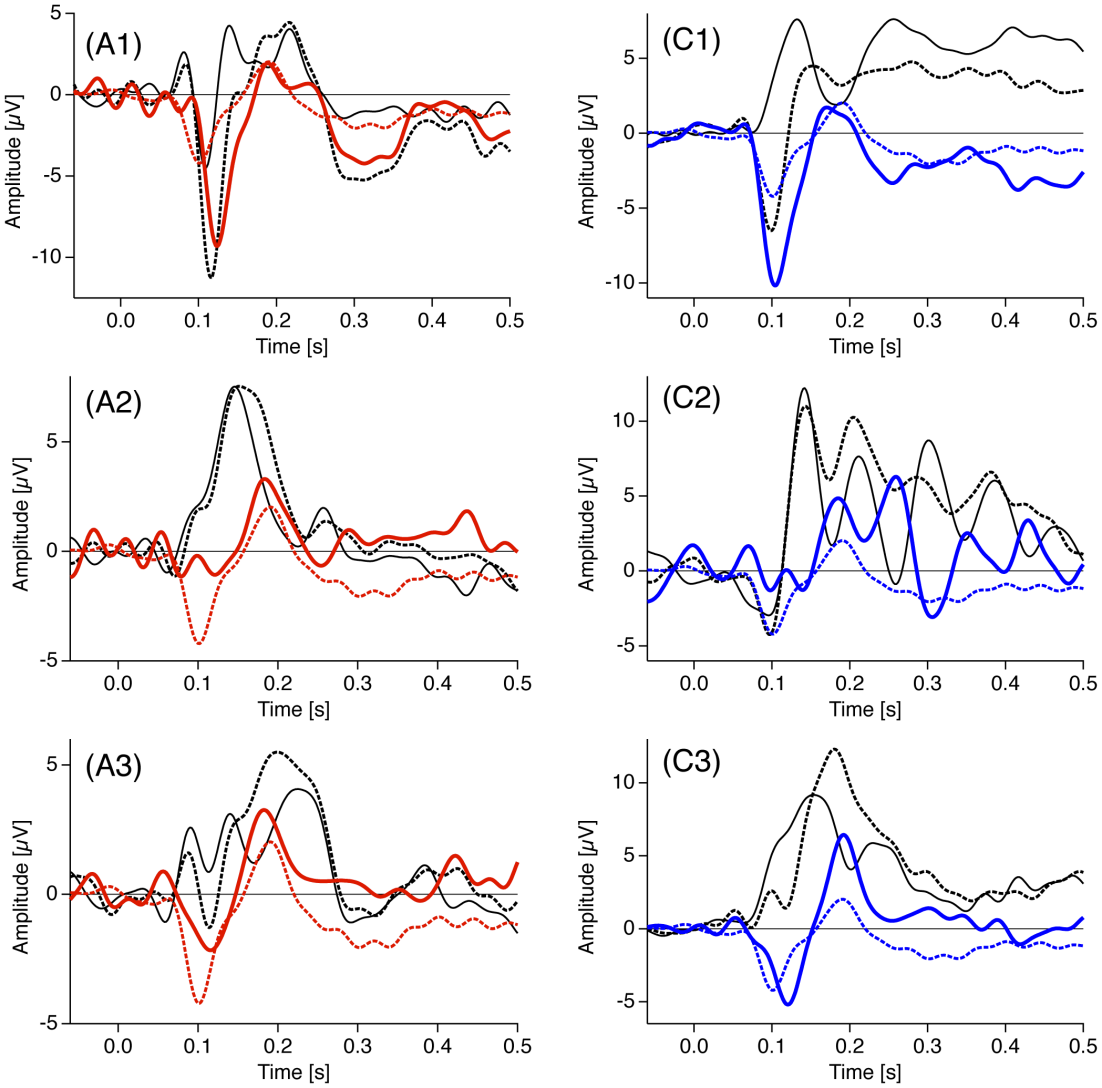
Individual examples of the ERP checksize effect at electrode O1. Black traces represent the raw ERP traces for fine (dashed traces) and coarse (continuous traces) checkerboards. Bold red (Asperger, “A”) and bold blue (Control observers, “C”) continuous traces show the individual differences (dERPs, fine minus coarse checkerboards). The red dashed traces represent the grand mean dERPs. The individual difference traces are less variable than the raw ERP traces (thus more similar to the grand means) but some variability remains. The chosen examples show observers with the effect mainly at the negativity (A1 and C1), mainly at the subsequent positivitiy (A2 and C2) or on both components (A3 and C3). All graphs are scaled for individual maxima and minima.

However, such inferences are most informative if they can be made on the level of single participants. Unfortunately, effects identifiable at the single participant level are rare because of the large inter-individual variability in general and particularly with the present data, as can be seen in Fig. 8 (black traces). We reduced the inter-individual variability in checkerboard ERP-responses considerably by calculating the difference between the ERPs to fine and coarse checkerboards (dERPs, blue and red traces in Fig. 8). The resulting pattern including a dERP negativity and a subsequent dERP positivity (blue and red dashed traces) is visible in all example participants but still with some inter-individual variance. Interpreting each single deflection in the context of autism-specific visual processing would necessitate to subdivide our participants into four groups with either an effect (1) mainly at the earlier negativity (Fig. 8 A1 and C1), (2) mainly at the later positivity (Fig. 8 A2 and C2), (3) at both components (Fig. 8 A3 and C3) and (4) a group without an evident effect. A cluster analysis, however, would need much more participants. It may be promising for future studies to test a broader range of spatial frequencies and therewith perhaps increase the discriminatory power of the ERP checksize effect. With optimal spatial frequencies at hand the basic paradigm would be very cheap, easy and fast and may be applied routinely in diagnostics to collect much more data and then do the above mentioned cluster analysis. In this context the following should be mentioned: The size of our coarse checkerboards (0.6° visual angle) can be translated to a spatial frequency of 1.18 cpd and is thus near to the critical spatial frequency of 2.2 cpd, which is reported to be important for face processing, e.g. [32], [33]. It has been shown, that autistic observers show differences in the visual processing of faces compared to normal controls [10]. These high-level differences in face processing, the here-presented lower-level differences in spatial frequency processing and the earliest visual differences cited above may be causally related. For a similar line of reasoning see [31]. Further research in this direction may be promising to understand functional relations.

### The Late P3b Effect

The P3b is one of the most prominent cognitive ERP components. It occurs with all sensory modalities and it seems to be composed by several neural processes around 300 ms after stimulus onset, e.g. [20]. It is labeled as cognitive because its amplitude is most dependent on the given experimental task and the frequency of occurrence (or absence) of a certain task-relevant stimulus and less on the lower-level stimulus features like size or spatial frequency, as can be observed in the present data. Interpretations of the functional role of the P3b range from context/working memory updating, e.g. [34], mediating between perceptual analysis and response initiation [35] or inhibitory processes during focused attention, e.g. [21]. The P3b has recently been discussed as one of the most reliable markers to distinguish unconscious (P3b absent) and conscious (P3b present) processing [19] although its generality and the large number of probably contributing brain sources make its relation to a specific cognitive function difficult, e.g. [20]. But see [36] for a more specific speculation.

Several P3b oddball studies have been performed with autistic and normal observers. The results are heterogeneous, with findings of smaller and larger amplitudes of the P3b in autistic observers compared to control observers or with no P3b difference at all. For reviews see [9], [11]. To our knowledge the evidence for an asymmetry of the P3b amplitude has only rarely been reported, e.g. [21], [37]. And we are particularly unaware of any report about differences in hemispheric symmetry of the P3b amplitude between autistic and normal observers.

One potential explanation of our P3b findings may be related to the observation that attention to the target stimulus is a necessary precondition for the P3b to occur: It is obvious and well documented that our attention system has limited capacities, e.g. [38]. There is evidence that this attention capacity is even more limited in certain subtypes of autism, like high functioning autism, e.g. [9]. It also might be possible that the attention capacity in AS is chronically overloaded by over-detailed and irrelevant information and more effort is necessary to deliberately change attention. Our data show a right-hemispheric lateralization of the P3b, which is more pronounced in the AS compared to control observers. It is well known that stimulus-induced attention capture also shows a right-hemispheric lateralization. And like the P3b this effect occurs across modalities, e.g. [39], [40]. Thus the degree of lateralization of the P3b may reflect – at least in the present paradigm – the amount of attentional resources employed in the task and may explain our finding of stronger lateralization in AS compared to the control observers. This speculation could be easily tested by studying the relation between P3b lateralization and attentional load in both AS and control observers (for attentional load paradigms see [41]). An optimal degree of task complexity may then increase the discriminatory power of this lateralization effect.

### Some Speculations on the Relation between the Early ERP Checksize and Late P3b Effects in Autism

Our main findings are that of a smaller checksize ERP effect and a more lateralized P3b effect in AS observers compared to controls. One might speculate that the smaller checksize ERP effect in the AS group represents alterations in low-level visual information processing in the primary occipital visual cortex of autistic observers. This might well relate to the common descriptions of altered visual experience of these people. Currently we do not know how to interpret smaller variance of the checksize effect for Asperger compared to control observers.

The lateralization of the P3b signal in the AS group might reflect the sequels of difficulties in early visual contextual information analysis which might result in a higher load of consciously driven top-down fronto-occipital analytical work load or work load of the global neuronal workspace system, e.g. [19]. Thus, the lateralization of the P3b signal might be seen as a compensatory consequence of the compromised early checksize signal in that higher degrees of conscious analytical information processing compensate for difficulties in low-level signal analysis. While speculative such interpretations relate well to clinical phenomenological observations and theoretical concepts. Clinically, particularly highly intelligent ASD-patients often report that they have learned to deliberately regulate their attention (i.e. to focus on social interaction) and are used to apply more conscious effort to focus on the requested task in a specific situation, which may be reflected by the stronger P3b lateralization. Theoretically, for example Shalom [42] has put forward the hypothesis that loss of or reduced integration of primary sensory stimuli in ASD might induce a higher degree of compensatory conscious analysis which in turn might cause other mental problems, like for example cognitive slowing and perceptual and/or attentional over-loads. These issues can be empirically tested in further research. A functional relation between the early ERP checksize effect and the later P3b lateralization effect should be reflected in their high correlation, which we did not find. The reason may be that early visual ERP responses and particularly onset responses are highly variable between participants, e.g. [16], [43]. This may be caused by large inter-individual variability in brain anatomy, e.g. cortical folding. Thus, similarities in functional processing not necessarily come along with similarities in surface EEG potentials. But optimizing stimulation parameters sometimes helps to increasing signal-to-noise ratio and thus increasing the number of participants that show both effects concurrently.

### Summary

The difference traces (dERPs) between fine and coarse checkerboards show a sizable effect of checksize (or spatial frequency) at occipital electrodes and thus probably in visual areas. This effect is smaller in AS observers compared to their matched controls with a discriminatory power of about 74%. Our findings fit well with recent results from the literature and even extend them from early visual steps around 100 ms to intermediate steps around 200 ms after stimulus onset. The fine checkerboards we used have a dominant spatial frequency close to the critical values for face processing, which is also known to differ between AS and normal observers. Potential causal relations between early visual and higher level specificities in AS and more general autistic observers may be identifiable in future experiments.

In addition to these lower-level visual effects we found a stronger right-hemispheric lateralization of the late P3b ERP component in AS than in control observers with a discriminatory power of about 75%. This P3b lateralization difference may be related to a difference in attentional resources between autistic and normal people. Combination of the two effects increases the discriminatory power to about 84%. For both effects it may be possible to increase effect sizes and thus the discriminatory power by optimizing checkerboard sizes (spatial frequencies) and task complexity (addressing attentional resources). In this case the two effects may be promising candidates for physiological markers in clinical diagnostics of Asperger’s syndrome and other autistic disorders.
